# Heterogenous profiles between primary lung cancers and paired brain metastases reveal tumor evolution

**DOI:** 10.3389/fonc.2023.1026099

**Published:** 2023-06-13

**Authors:** Yanming Chen, Xiaoxiao Dai, Ji Wang, Chuming Tao, Ye Wang, Qing Zhu, Zhongyong Wang, Tan Zhang, Qing Lan, Jizong Zhao

**Affiliations:** ^1^ Department of Neurosurgery, Second Affiliated Hospital of Soochow University, Suzhou, China; ^2^ China National Clinical Research Center for Neurological Diseases, Beijing Tiantan Hospital, Capital Medical University, Beijing, China; ^3^ Department of Pathology, Second Affiliated Hospital of Soochow University, Suzhou, China; ^4^ Health Management Center, Second Affiliated Hospital of Soochow University, Suzhou, China; ^5^ Department of Neurosurgery, Beijing Tiantan Hospital, Capital Medical University, Beijing, China

**Keywords:** heterogeneity, brain metastasis, lung cancer, gene mutation, evolution

## Abstract

**Background:**

Brain metastases (BMs) are the most common central nervous system (CNS) malignant tumors, with rapid disease progression and extremely poor prognosis. The heterogeneity between primary lung cancers and BMs leads to the divergent efficacy of the adjuvant therapy response to primary tumors and BMs. However, the extent of heterogeneity between primary lung cancers and BMs, and the evolutionary process remains little known.

**Methods:**

To deeply insight into the extent of inter-tumor heterogeneity at a single-patient level and the process of these evolutions, we retrospectively analyzed a total of 26 tumor samples from 10 patients with matched primary lung cancers and BMs. One patient underwent four times brain metastatic lesion surgery with diverse locations and one operation for the primary lesion. The genomic and immune heterogeneity between primary lung cancers and BMs were evaluated by utilizing whole-exome sequencing (WESeq) and immunohistochemical analysis.

**Results:**

In addition to inheriting genomic phenotype and molecular phenotype from the primary lung cancers, massive unique genomic phenotype and molecular phenotype were also observed in BMs, which revealed unimaginable complexity of tumor evolution and extensive heterogeneity among lesions at a single-patient level. By analysis of a multi-metastases case (Case 3) of cancer cells’ subclonal composition, we found similar multiple subclonal clusters in the four spatial and temporal isolated brain metastatic focus, with the characteristics of polyclonal dissemination. Our study also verified that the expression level of immune checkpoints-related molecule Programmed Death-Ligand 1 (PD-L1) (P = 0.0002) and the density of tumor-infiltrating lymphocytes (TILs) (P = 0.0248) in BMs were significantly lower than that in paired primary lung cancers. Additionally, tumor microvascular density (MVD) also differed between primary tumors and paired BMs, indicating that temporal and spatial diversity profoundly contributes to the evolution of BMs heterogeneity.

**Conclusion:**

Our study revealed the significance of temporal and spatial factors to the evolution of tumor heterogeneity by multi-dimensional analysis of matched primary lung cancers and BMs, which also provided novel insight for formulating individualized treatment strategies for BMs.

## Introduction

Brain metastases (BMs) represent the most common type of malignant tumor in the central nervous system (CNS) (1). BMs most commonly originate from lung cancer (2). The incidence of BMs has been increasing in recent years; due to the accumulated advances in the diagnostic and therapeutic strategies for primary lung cancers (3, 4). Patients who progressed to BMs always suffered significantly worse prognostic outcomes (5). The median overall survival time of patients with BMs left a dismal 5 weeks if untreated, while this can be prolonged to 3-18 months with modern treatment modalities (6).

Immune checkpoint inhibitors have become research focuses currently (7). However, accumulating evidence suggests that the special CNS microenvironment for metastatic tumor cells and the heterogeneity between primary tumors and BMs during the evolution often become critical obstacles leading to treatment failure (6, 8). The heterogeneity of BMs represents a severe challenge to precision medicine for BMs, which becomes an obstacle to the individualized treatment of BMs (9). To date, the extent of heterogeneity between BMs and primary tumors remains controversial, and the evolution of heterogeneity between BMs and primary tumors is largely unknown (10, 11). The extensive heterogeneity and tumor evolution within primary tumors also lead to a question as to whether metastasis is monoclonal dissemination or polyclonal dissemination, which is still controversial (12, 13).

During the process of BMs formation, the shared molecular mechanism of the primary tumors plays its role, as well as the influence of CNS microenvironment on the disseminated tumor cells. It is hard to evaluate which side plays a prominent role. Though the long-standing dogma of “immune privilege” in CNS has been reconsidered in recent years (14), its high immunosuppressive characteristic still being a serious obstacle to current tumor immunotherapy. In the past few years, immune checkpoint inhibitors have achieved great success in the treatment of lung cancer. However, the effect of immune checkpoint inhibitors on BMs remains controversial (15), and some clinical trials which included patients with BMs are underway. It has been reported that the expression level of PD-L1 in melanoma BMs was significantly lower than that of the primary tumors and extracranial metastases (6). This variability of PD-L1 expression and tumor-infiltrating lymphocytes (TILs) density between primary lung cancers and paired BMs may account for the variability in response to immune checkpoint inhibitors.

In this study, we comparatively analyzed the data of capture-based WESeq and immunohistochemical profiles to assess the genomic heterogeneity, tumor driver genes, and immune molecules variability between the primary lung cancers and paired BMs, based on paired analysis of a cohort of Primary-BMs samples. We expect deep insight into the extent of heterogeneity between primary lung cancers and BMs, and to explore the genomic and immunophenotypic heterogeneity evolutionary trajectory with multiple BMs that occurred at different spatial and temporal points at a single-patient level. Our study also provides new insights into the individualized treatment of BMs and gives us novel clues to the controversy over the model of metastatic dissemination.

## Materials and methods

### Patient cohorts

Ten patients from the Second Affiliated Hospital of Soochow University (Suzhou, China) were included in our retrospective study from 2013 to 2021, with matched primary lung tumors and BMs. A total of 26 tumor paraffin-embedded specimens were collected. As shown in **Table 1**, demographic and clinical information, including age at cancer diagnosis, gender, overall survival time, pathology, and adjuvant therapy was abstracted from each patient’s medical record. This study was conducted following the ethical principles set forward in the Declaration of Helsinki. All patients provided written consent to participate in this analysis study.

**Table 1:** The clinical data of primary lung cancers and paired BMs.

Case No.	Exp.ID	Age (Years)^1^	Gender	Metastatic lesion Location	Interval time (Months)^2^	Treatment of primary lesion	Treatment of metastatic lesion	Pathology (Type)		Molecular characteristics^3^		OS(Months)	Adjuvant therapy^4^	End
								Primary	Metastatic	Primary	Metastatic			
1	LC_001	60	M	Lt. Cerebellum	-35	Biopsy	Total resection	Poorly differentiated SC	AC	EGFR, PD-L1	EGFR, PD-L1	38	RT	D
2	LC_002	58	M	Rt. Temporal lobe	11	Resection	Total resection	Poorly differentiated SC	SC	EGFR, PD-L1, ALK	EGFR, PD-L1, BRAF	72	PTX and Ox	D
3	LC_003	57	M	Rt. Occipital lobe	-2	Resection	Total resection	ASC	AC	EGFR, PD-L1, ROS1	EGFR, PD-L1, ROS1	54	NC	D
	LC_004	58		Lt. Occipital lobe	8		Total resection		AC		EGFR, PD-L1, ROS1, ALK		PTX and Ox	
	LC_005	60		Rt. Cerebellum	31		Total resection		AC		EGFR, PD-L1		-	
	LC_006	61		Lt. Cerebellum	47		Total resection		AC		EGFR, PD-L1, ROS1		-	
4	LC_007	44	F	Rt. Temporal parietal lobe	-15	Biopsy	Total resection	AC	Poorly differentiated AC	EGFR, PD-L1	EGFR, PD-L1	69	NC	A
5	LC_008	61	M	Lt. Frontal lobe	8	Resection	Total resection	Poorly differentiated AC	AC	EGFR, PD-L1, BRAF	EGFR, PD-L1, ROS1	41	Pem, NDP and RT	A
6	LC_009	38	F	Subclavian lymph node	0	Resection	Biopsy	AC	AC	EGFR, PD-L1, ALK	–	19	NC	D
	LC_010	39		Lt. Frontal lobe	11		Total resection		AC		EGFR, PD-L1, ALK		IH	
	LC_011	39		Lt. Thalamus	11		Total resection		AC		EGFR, PD-L1, ALK		IH	
	LC_012	39		Subclavian lymph node	14		Biopsy		AC		–		IH	
7	LC_013	58	M	Lt. Occipital lobe	-0.5	Resection	Total resection	Poorly differentiated AC	AC	EGFR, PD-L1, RET	EGFR, PD-L1, RET	23	Anl+PD1 inhibitor	A
8	LC_014	63	M	Rt. Occipital lobe	22	Resection	Total resection	AC	AC	EGFR, PD-L1	EGFR, PD-L1	30	NC	D
9	LC_015	42	M	Rt. Frontal temporal lobe	60	Resection	Total resection	Poorly differentiated AC	AC	EGFR, PD-L1	EGFR, PD-L1	110	PTX and Ox	D
10	LC_016	68	F	Lt. Frontal parietal lobe	47	Resection	Total resection	AC	AC	EGFR, PD-L1, ALK	EGFR, PD-L1, ALK	67	Cri	A

1 The age of onset, regardless of whether the primary lesion diagnosis prior, or earlier diagnosis of metastases.

2 The value is positive, if treatment of the primary lesion prior to metastases, otherwise the value is negative.

3 Positive molecular characteristics (EGFR, immunohistochemistry positive rate of more than 20% or deleterious mutant; PD-L1, immunohistochemistry positive rate of more than 5%; ALK, immunohistochemistry ALK(D5F3) positive or deleterious mutant; BRAF, ROS1 and RET, all deleterious mutant).

4 Preoperative treatment of brain metastases.

OS, Overall survival; ASC, Adenosquamous carcinoma; AC, Adenocarcinoma; SC, Squamous carcinoma; RT, Radiotherapy; PTX, Paclitaxel; Ox, Oxaliplatin; Pem, Pemetrexed; NDP, Nedaplatin; IH, Icotinib Hydrochloride; Anl, Anlotinib; Cri, Crizotinib; D, Dead; A, Alive,

### Whole exome sequencing and data processing

Genomic DNA from FFPE samples was extracted with GeneRead DNA FFPE Kit (180134, Qiagen, Germany). Genomic DNA samples were captured using the Agilent SureSelect Human All Exon v6 library following the manufacturer’s protocol (Agilent Technologies, USA). Briefly, approximately 3μg genomic DNA was sheared to 150 to 220bp small fragments using a sonicator (Covaris, Inc., USA). The sheared DNA fragments were purified, adapters from Agilent were ligated onto the polished ends and the libraries were amplified by polymerase chain reaction (PCR). The amplified libraries were hybridized with the custom probes. The DNA fragments bound with the probes were washed and eluted with the buffer. Then these libraries were sequenced on the Illumina sequencing platform (HiSeq X-10, Illumina, Inc., USA), and 150bp paired-end reads were generated.

The raw reads were pre-processed with fastp (Version 0.19.5). Clean reads were aligned to the reference human genome (GRCh37) utilizing the BWA (Version 0.7.12). The mapped reads were sorted and indexed with SAMtools (Version 1.4). GATK (Version 4.1.0.0) was utilized for recalibration of the base quality score and single nucleotide polymorphism (SNP) and insertion/deletion (InDel) realignment. The frequency of SNP in 1000 Genomes Project or the Genome Aggregation Database (gnomAD) > 1% subpopulation was excluded. Copy number variation (CNV) was inferred from sequencing data using the software package CNVkit (Version 0.9.5), and Lumpy software (Version 0.2.13) was applied to call structural variation (SV). The detected genomic variation information was visualized using the Circos diagram. For shared mutations between primary tumors and brain metastatic tumors within each patient, we considered only ‘D’ level mutations of SIFT (Sorts Intolerant From Tolerant) and Polyphen2_HDIV (Polymorphism Phenotyping v2) evaluation.

### Histopathological analysis

All slices from the primary tumors and BMs of this cohort were independently diagnosed by two experienced pathologists. The immunohistochemical staining of EGFR (SP125, VENTANA, USA), PD-L1 (SP263, VENTANA, USA), CD34 (Kit-0004, MXB, China), CD4 (RMA-0620, MXB, China), CD8 (RMA-0514, MXB, China), ALK (D5F3, VENTANA, USA) of FFPE followed the protocol. Microvascular density (MVD) of tumor tissue was assessed by a method published by Weidner (16).

### Statistical analysis

GraphPad Prism (Version 8.0) was used for data analysis. Images were analyzed and recorded with Fiji (NIH open access, USA). The mean value differences were compared by analysis of variance (ANOVA). A paired Student’s t-test was used to identify the difference between groups. A P-value < 0.05 suggested statistical significance.

## Results

### Clinical characteristics of patients with primary lesions and paired BMs

**Figure 1:**
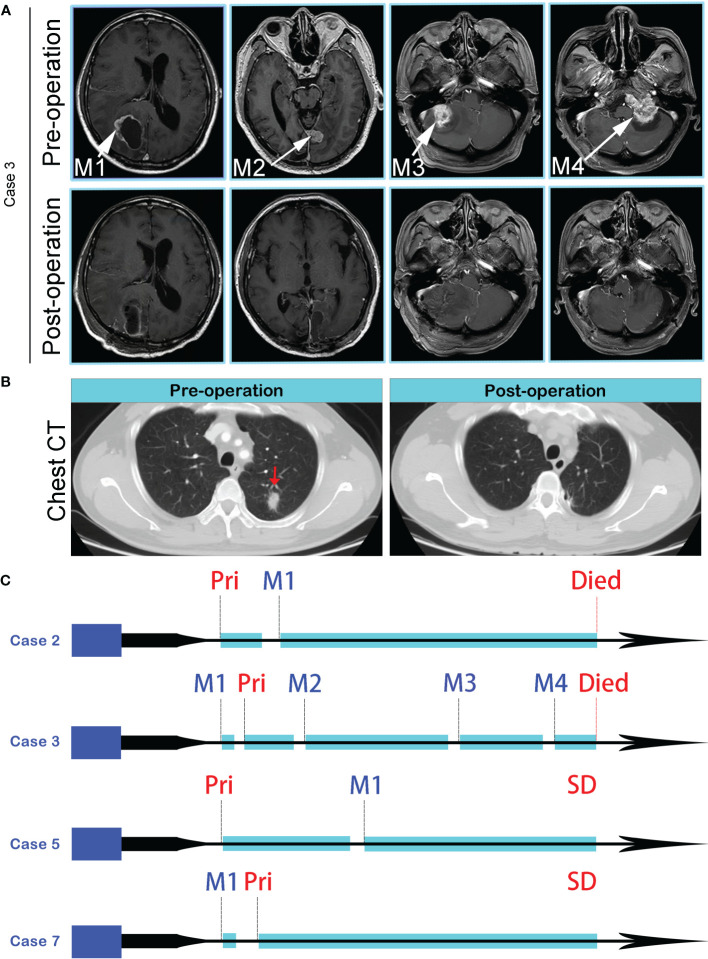
**(A)** MR images of four spatial and temporal isolated BMs in Case 3, before (the upper panel) and after (the lower panel) surgeries. **(B)** CT imaging revealed an isolated lung lesion (Red arrow), and postoperative chest CT showed the lesion was removed totally. **(C)** Disease progression and treatment timelines of Case 2, Case 3, Case 5, and Case 7. Pri, Primary lung tumor; SD, Stable Disease.

10 primary lung cancer patients with BMs were included in this study (**Table 1**). All patients received primary tumors and BMs surgeries, and all tumor specimens were available. Therein, Case 3 underwent 4 times BMs surgeries within a time spanning up to 54 months (**Figure 1**), and two isolated brain metastatic specimens were obtained from Case 6. A cohort of 26 tumor specimens was analyzed (number of primary tumors and BMs, 10 vs. 16). The median overall survival time diagnosed with BMs was 35.5 months (range, 8-69 months). Recent studies implicated that the dissemination of primary tumors can occur at every stage of tumor progression (11). This cohort of cases also supported the perspective. Part of the cases (4/10) developed disseminated BMs at the very early stage of lung cancer (**Table 1**). Those 4 patients first manifested the symptom of headache or neurological deficit. The 4 patients underwent surgery for BMs and were followed up by receiving primary lesions surgery, with interval times 0.5 to 35 months. The remaining 6 patients all received the primary lesion surgery, and BMs emerged at different intervals when patients manifested as CNS clinical presentation, and the surgery of BMs resection followed. Additionally, there were no more than 3 intracranial isolated metastatic lesions simultaneously, when deciding to perform BMs surgery.

### Genomic heterogeneity analysis of primary tumor and paired BMs

**Figure 2:**
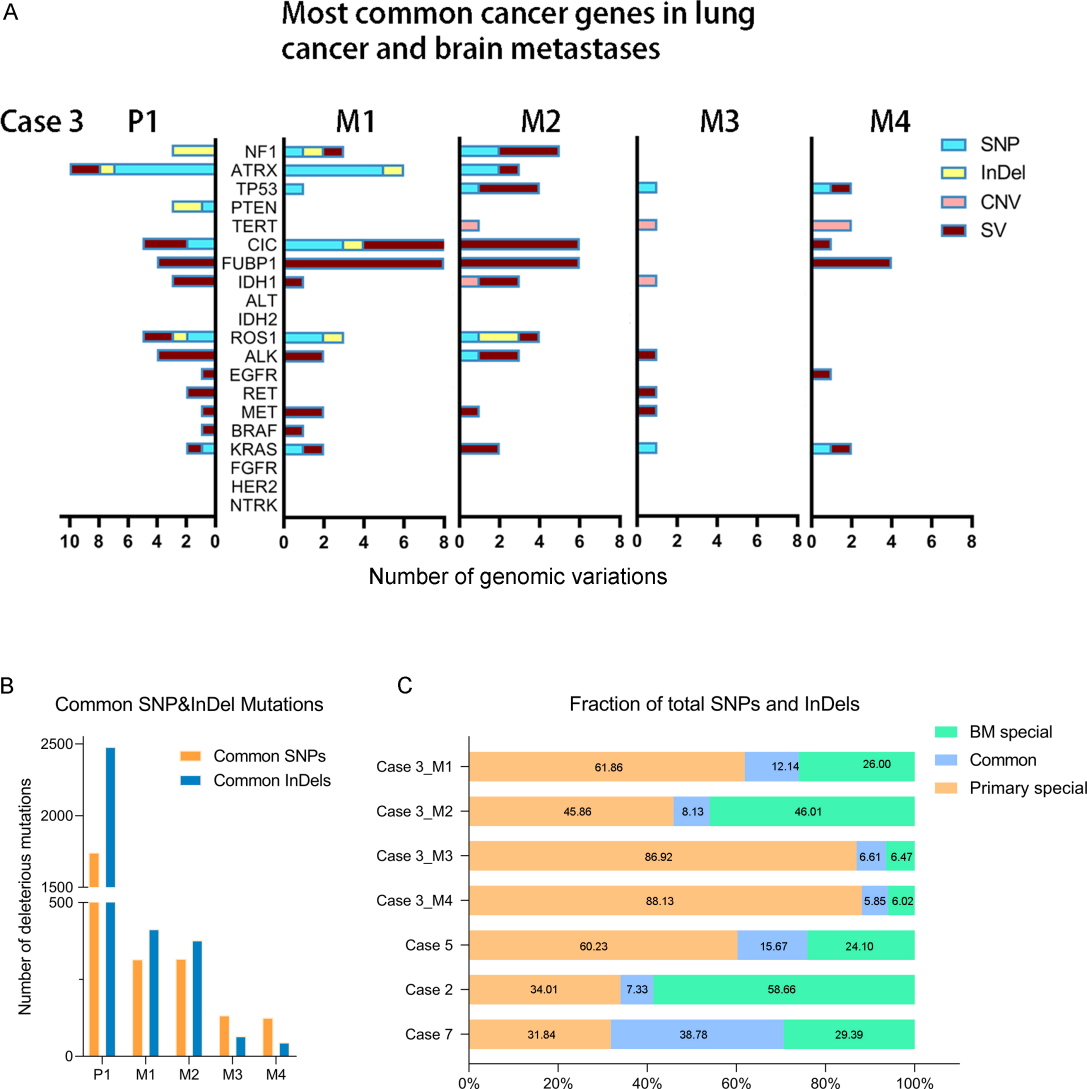
**(A)** The distribution of common lung cancer- and brain tumor-associated cancer gene genomic variations in primary lung tumor and different BMs of Case 3. **(B)** The distribution of the common SNP and InDel mutations between primary lung cancer and four BMs in Case 3. **(C)** The shared deleterious SNP and InDel may be varied between the primary lung cancer and paired BMs.

**Figure 3:**
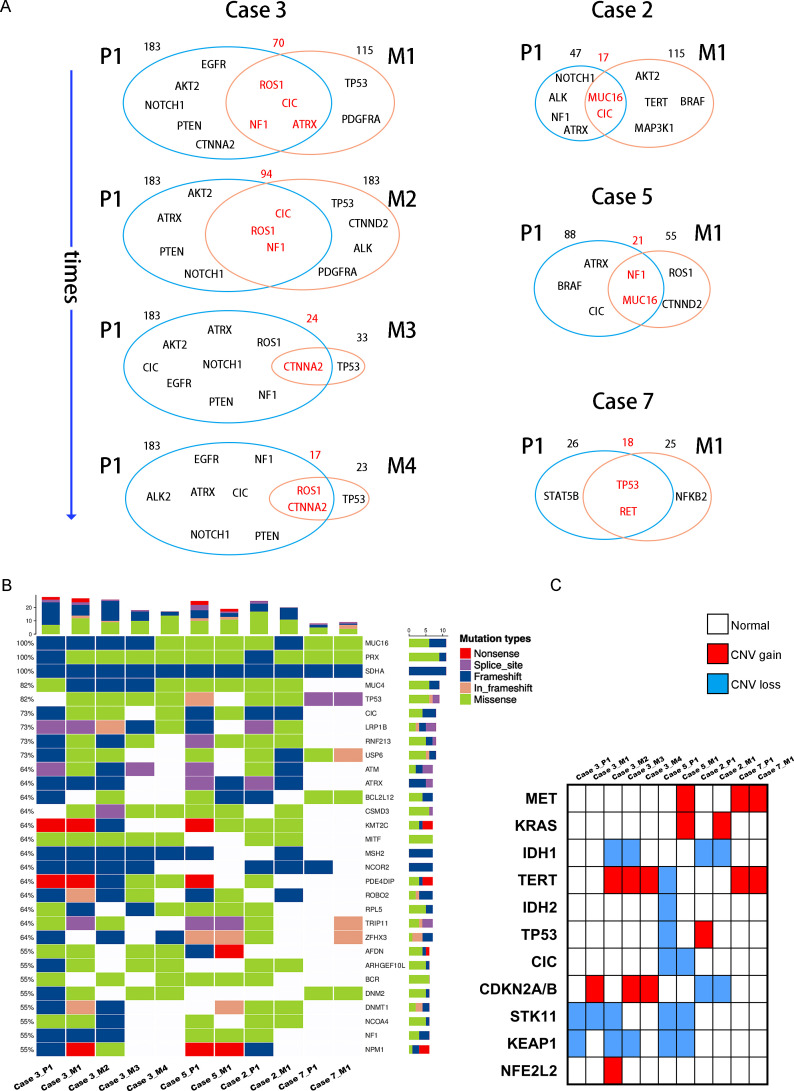
**(A)** Venn diagram exhibits the genetic concordance (SNP and InDel) between the primary lung tumors and paired BMs. **(B)** Heatmap of the top 30 common tumor driver genes mutations. **(C)** Heatmap shows common lung cancer- and brain tumor-associated cancer genes copy number variation in the primary lung tumors and paired BMs.

We completed a cohort of 11 primary lung tumors and paired BMs tumor tissues WESeq (Case 2, Case 3, Case 5, and Case 7) (**Figure 1C**). To assess the genomic variability, the genomic SNPs and InDels of the paired primary and brain metastatic lesions were compared. Consistent with the previous report (17), primary tumors accumulated more genomic variation than BMs (Case 2, Case 3, Case 5, and Case 7) (**Supplementary Figure 1A, B**). Subsequently, we screened the mutation sites for deleterious and compared the primary lesion with 4 isolated brain metastatic lesions in Case 3. We found that SNPs (including missense, stopgain, and stoploss) and InDels (including framesShift, stopgain, and stoploss) shared a remarkable diversity between the primary lesion and the four spatial and temporal isolated BMs (**Figure 2A**). The primary tumor accumulated more deleterious genomic variations (SNP and InDel) than the 4 spatial and temporal isolated BMs in Case 3 (**Figure 2B**), and the shared SNPs and InDels between primary lung cancer and BMs gradually decreased as the disease advanced (**Figure 3A**). We also found that compared with the primary tumor, BMs only inherited a fraction of SNPs and InDels (Case 2, Case 3, Case 5, and Case 7; ranging from 5.85% in Case 3_M4 to 38.78% in Case 7) (**Figure 2C**). **Figure 3B** summarized the frequency spectrum of common driver gene mutations. MUC16, PRX, and SDHA showed the highest frequency of deleterious mutations, occurring in all primary lung cancers and BMs (Case 2, Case 3, Case 5, and Case 7).

**Figure 4:**
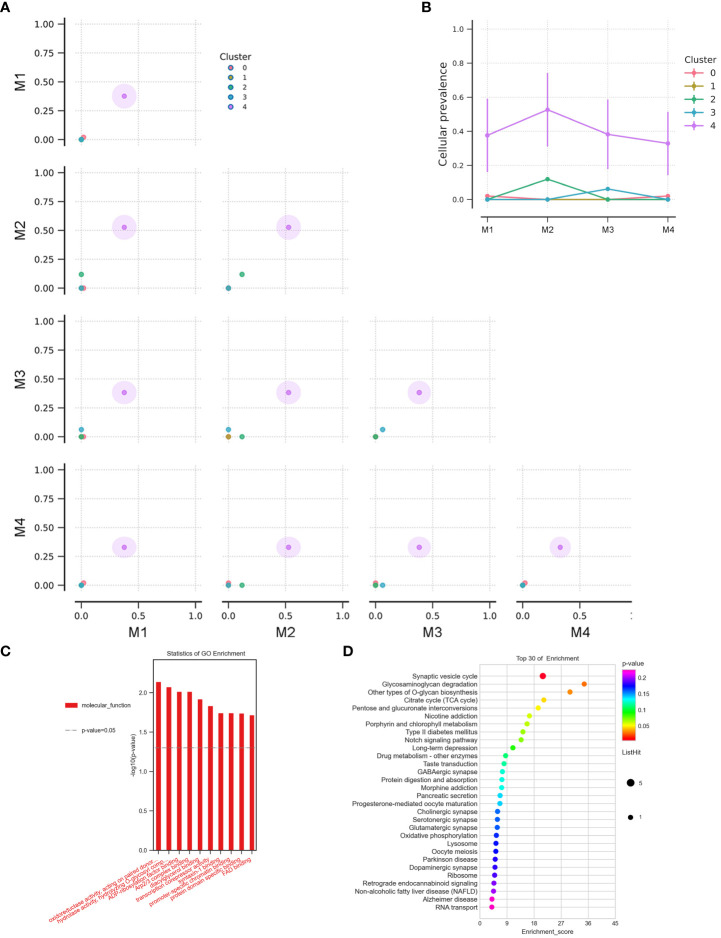
**(A)** The cluster correlation between samples of BMs in Case 3. **(B)** The cellular prevalence of each cluster in the BMs of Case 3. GO functional **(C)** and KEGG pathway **(D)** enrichment analysis of the common mutant genes in the BMs (Case 2, Case 3, Case 5, and Case 7).

Additionally, CDKN2A/B genes, a set of recognized lung cancer suppressor genes, copy number amplification was observed in M1, M3, and M4 BMs of Case 3, while no aberrations were found in the primary tumor and M2 (**Figure 3C**). Our study also indicated that STK11 and KEAP1 copy number deletions occurred in Case 3 and Case 5, but not all BMs in Case 3 were consistent (**Figure 3C**). Moreover, CNV events of a cohort of genes, including KRAS, CDKN2A/B, and IDH1, were frequently found in brain metastatic lesions (**Figure 3C**). Integrated analysis of the genomic variation of the patient’s primary tumors and BMs, we found that the genomic heterogeneity between the primary tumors and BMs was striking higher than we expected (**Figure 3**, **Supplementary Figure 2**). The Circos diagram revealed that Case 3_M2 showed a significantly higher frequency of chromosomal aberrations (**Supplementary Figure 2B**), such as interchromosomal translocations. The analysis of cancer cells’ subclonal composition in BMs revealed similar subclonal cluster composition in Case 3, with the characteristics of polyclonal dissemination (**Figures 4A, B**).

To better comprehend the function of deleterious mutant genes, GO functional and KEGG pathway enrichment analyses were performed. GO enrichment analysis indicated that the mutant genes in BMs were more activated in molecular functions related to oxidoreductase activity and hydrolase activity (**Figure 4C**). KEGG enrichment analysis of the shared mutant genes in the BMs found that the mutant genes were mainly enriched in glycosaminoglycan degradation (**Figure 4D**). **Supplementary Table 1** exhibits the unique genetic signatures in BMs, and TP53 mutation frequently occurs in BMs, although the impact of TP53 mutation on the advantage of cancer cells’ brain dissemination is still unclear.

### Internal histopathologic heterogeneity and evolution analysis

**Figure 5:**
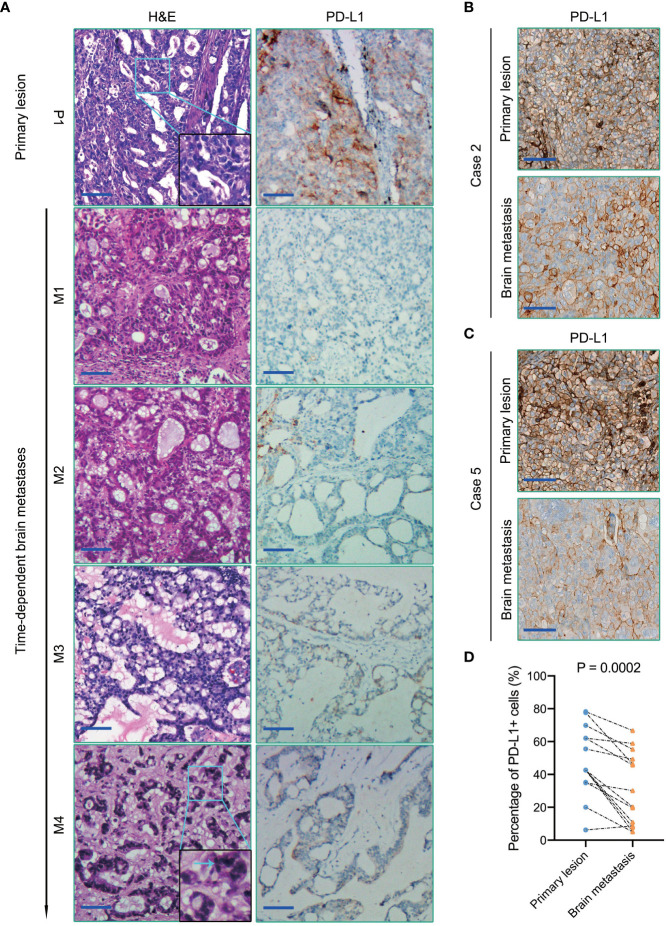
**(A)** H&E staining (the left panel) and PD-L1immunohistochemical staining (the right panel) of primary lung cancer and BMs in Case 3. The blue arrow in the magnified image points to the nucleus with apparent atypia. **(B, C)** PD-L1 immunohistochemical staining of primary lung cancers and BMs in Case 2 and Case 5, respectively. **(D)** Quantification of PD-L1 expression in each primary lung tumor and paired BMs. Scale bar = 100μm.

In most cases, the histomorphological differences between the primary lung tumors and BMs were limited (**Figure 5A**, **Supplementary Figures 3B-D**). However, we still observed some alterations in Case 3 with multiple recurrences. From foci M1 to M4, it showed a variable level of cancer cell differentiation, from well-differentiated to poorly differentiated. Metastatic lesions still basically maintained the histopathological characteristics of the primary lung cancer at an early stage of dissemination. However, we found a remarkably poorer degree of tumor differentiation in the extremely long-term brain metastatic foci than the primary lung cancer, with the classic tube-like structure gradually disappearing. Tumor cell clusters were diffusely distributed, and the nucleus atypia, mitosis, and giant polymorphic nucleus could be observed frequently in metastatic foci M4 (**Figure 5A**).

### Tumor-related immune heterogeneity between primary lesions and paired BMs

Emerging evidence suggests that the brain is not an “immune privileged” organ as previously thought. The immune checkpoint therapy targeting the PD1/PD-L1 pathway has notably improved the survival outcomes of several types of malignant tumors, which also brought hope to patients with BMs. Higher PD-L1 expression of tumor cells and/or TILs density is always associated with favorable anti-PD1/PD-L1 immunotherapeutic efficacy (18). However, in contrast with the primary lesions, the efficacy of immune checkpoint therapy targeting PD1/PD-L1 in BMs is indeed not so significant (19, 20). Herein, we assessed PD-L1 expression in these ten primary lung cancers and paired BMs patients by immunohistochemistry. Our study indicated that the expression level of PD-L1 in brain metastatic tumor cells was significantly lower than that of paired primary lung cancers (P = 0.0002) (**Figure 5**).

**Figure 6:**
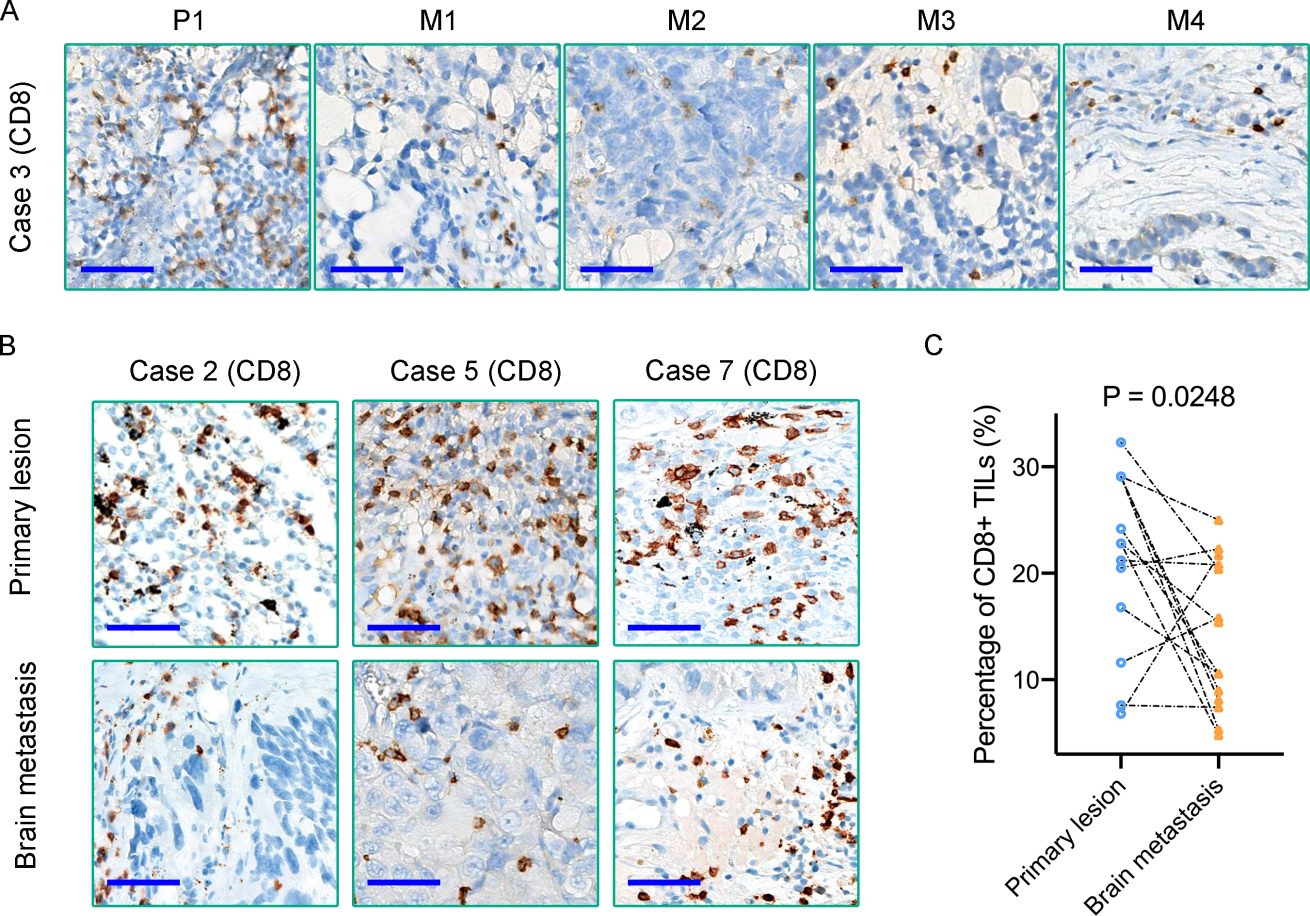
**(A, B)** Immunohistochemical staining reveals the density of CD8+ TILs in matched primary lung tumors and BMs; **(C)** Quantification of CD8+ TILs density in each primary tumor and paired BMs. Scale bar = 50μm.

TILs, especially CD8+ TILs, represent a favorable prognostic factor in several types of cancers (21). Meanwhile, CD8+ TILs performed as the final executor of PD1/PD-L1 pathway. A comparative study of these ten primary tumors and paired metastases patients revealed that the density of CD8+ TILs in BMs was significantly lower than that of matched primary tumors (P = 0.0248) (**Figure 6A–C**). Our results also demonstrated a relatively lower density of CD4+ T cells in primary lung cancers (Case 2, 3, 5, and 7) (**Supplementary Figure 4A, B**). Although only Case 7 was treated with PD1/PD-L1 blockade immunotherapies in this cohort of retrospective study, the above analysis still benefits us to comprehensively insight into the immune microenvironment of BMs and provides us novel clues to evaluate the efficacy of immunotherapy of BMs. Additionally, MUC16 was observed high frequency mutated in this cohort of cases (**Figure 3B**). CIBERSORT-based (TIMER2.0 (http://timer.comp-genomics.org/)) immune cell infiltration analysis found that the density of CD8+ T cells infiltrated in the MUC16 mutant subgroup was significantly higher than that in the MUC16 wild-type subgroup (P = 0.011) (**Supplementary Figure 4C**). In summary, BMs differed from paired primary tumors by showing more notable immunosuppressive characteristics than primary tumors.

### Analysis of immunohistochemical phenotypic heterogeneity between primary lesions and paired BMs

Finally, we integrative analyzed the molecular phenotypes of all 10 paired primary lung cancers and BMs. Although primary lung cancers and paired BMs shared pivotal molecular phenotypes, such as the EGFR expression and EML4-ALK fusion status in lung cancer cases (**Supplementary Figure 3A, B**, **Supplementary Table 2**). While comparatively analyzed tumor microvascular density (MVD) (CD34 staining), a notable difference in MVD was found between the primary lung cancers and paired BMs, and inter-BMs, with tumor MVD more plentiful in BMs than in the paired primary tumors (**Supplementary Figure 5**).

## Discussion

Traditionally, the occurrence of BMs always represents the end-product of tumor progression, but some researchers believe that the dissemination of primary tumor cells can occur at various stages of tumor progression (11, 22). Our result from a cohort of matched primary lung cancers and BMs cases tended to support the latter perspective, a proportion of patients (4/10) developed disseminated brain tumors before primary tumor resection, or parallel with primary tumor evolution. Although we have been warned that intra-tumors genetic heterogeneity was extensive, has long been believed that the phenotypic heterogeneity may not be so extensive, especially between the primary and metastatic lesions at a single-patient level. We ignored that, though the shared molecular mechanism in primary tumors played an important role, the influence of CNS microenvironment exerted on the disseminated tumor cells during BMs formation was equally vital (8).

Herein, based on the WESeq data, we first exhibited the deleterious genomic SNPs and InDels evolution landscape from a cohort of matched primary lung cancers and BMs. The results demonstrated that the shared mutations of lung cancer driver genes between the primary lung cancers and the BMs gradually decreased as time goes on, though the difference lacked statistical significance due to the limited number of cases. Subsequent CNV analysis of lung cancer- and brain tumor-associated driver genes revealed that there was also widespread heterogeneity between the primary tumors and BMs, even without a shared CNV event in the multi-metastases case (Case 3). CDKN2A/B, STK11, and KEAP1 had a high frequency of alterations in lung cancer BMs cases. STK11 and KEAP1 were considered to be associated with poor prognosis in patients diagnosed with lung adenocarcinoma (23). Noteworthy, the alterations of CDKN2A/B in three BMs showed copy number amplification in Case 3, instead of frequency CNV deletion reported (24, 25) in primary glioblastoma. The emergence of PDGFRA mutation in M1, and M2 may account for higher angiogenic phenotype, which may be a benefit for the disseminated cells’ brain metastases. It has been reported that the typical tumor marker CA125 encoded by the MUC16 gene played a vital role in regulating tumor cell metastasis (26), and succinate dehydrogenase (SDH) complex subunits mutations were reported highly associated with tumor cells metastases in pheochromocytoma and paraganglioma and other malignancies (27, 28). In this study, we found that MUC16, PRX, and SDHA showed a high frequency of deleterious mutations in all primary lung cancers, and BMs inherited the genomic variations, which indicated those genes may act as lung cancer cell dissemination driver genes. ROS1 and RET alterations are frequently observed in BMs, and the fusion or mutation can activate downstream MAPK/ERK, PI3K/AKT signaling pathways, which have been reported to play important roles in the development of brain metastases (29).

GO and KEGG analysis of the shared mutant genes in the BMs indicated that the mutant genes in BMs were more activated in metabolic activity functions and glycosaminoglycan degradation pathway. It has been reported that glycosaminoglycan biosynthesis and degradation are crucial for lung cancer epithelial-mesenchymal transition (EMT), which contribute to cancer cell metastasis by changing intracellular iron metabolism (30). For the particularity of brain metabolism, enhanced TCA-cycle and SDHA mutation may be a benefit in the disseminated cell clone formation in the brain microenvironment. Metabolic reprogramming, especially the glycosaminoglycan biosynthesis pathway, might contribute to lung cancer progression and metastasis (31), which may be a potential therapeutic target for the procession of lung cancer brain metastases. When regarding the origin of metastasis, there have always been two hypotheses, monoclonal dissemination and polyclonal dissemination from primary tumors (11). Echoing previous studies (32, 33), by analysis of a multi-metastases case (Case 3) of cancer cells’ subclonal composition, we tended to support the hypothesis of polyclonal dissemination.

In all types of metastatic tumors, a common feature in each step of tumor cell dissemination is the need to escape recognition and destruction by the immune system (6). This immune escape mechanism plays a vital role in the formation of BMs. We found that PD-L1 was highly expressed in primary lesions, while the expression of PD-L1 in brain metastatic lesions was significantly lower than that of matched primary lung tumors. Moreover, our study also found that the density of CD8+ TILs in BMs was remarkably lower than in matched primary lung cancers. All our results suggested that BMs showed the characteristic of immunosuppression. This clue also partly confirmed the current dilemma of immunotherapy of BMs. Immune checkpoint inhibitors seem to be less effective on BMs than on primary tumors (7, 34). The causes of extensive immune-associated phenotypic heterogeneity between primary tumors and BMs may vary (17). One possible factor, that the unique immunosuppressive characteristics of CNS microenvironment may shape clonal metastatic cancer genome evolution, cannot be ignored (11, 35). However, our study also has some limitations. First, due to a retrospective study, matched germline DNA as normal controls were not available, and lack of more suitable samples for further transcriptomic and proteomic analysis, which may provide more accurate details for the evolution of BMs heterogeneity. Additionally, restricted by the number of matched primary tumors and BMs, the statistical analysis of results is difficult. Hence, we need to accumulate more detailed matched primary lung cancer and BM cases in further studies, by combining with single-cell sequencing, to deeply analyze the role of the CNS microenvironment in shaping BMs.

Looking ahead, immunotherapy of BMs is still an important strategy, although immunosuppressive characteristics of the CNS microenvironment. Nevertheless, surgical resection of BMs is also of great significance, for giving the possibility of analyzing the unique immune characteristics of BMs and lending credence to individualized BMs immunotherapy consultation. To significantly boost the efficacy of BMs immunotherapy, we suspect that remodifying the immune microenvironment of BMs may be a promising approach.

## Conclusion

In summary, our retrospective analysis results shed light upon the significant heterogeneity between matched primary lung cancers and BMs, and the complexity of the evolutionary process, especially the evolutionary process of heterogeneity due to temporal and spatial dynamic changes at a single-patient level. And our results verified the characteristic of immunosuppressive in BMs, which also provide novel insight for formulating individualized treatment strategies for BMs.

## Data availability statement

The data presented in the study are deposited in the NCBI repository, accession number PRJNA733235.

## Ethics statement

The studies involving human participants were reviewed and approved by the ethics committee of the Second Affiliated Hospital of Soochow University. The patients/participants provided their written informed consent to participate in this study. Written informed consent was obtained from the individual(s) for the publication of any potentially identifiable images or data included in this article.

## Author contributions

YC designed this study. XD and CT were in charge of pathological diagnosis and immunohistochemical experiments. JW, QZ, YW, ZW, and TZ supported and analyzed clinical data. JZ and QL guided this work and reviewed the manuscript. All authors contributed to the article and approved the submitted version.

## Supplementary material

Supplementary Figure 1
**(A)** The distribution of various sites of SNP&InDel mutations in primary lung cancers and BMs; **(B)** Overall distribution of mutation types.

Supplementary Figure 2
**(A)** Landscapes of copy number variation. **(B)** Circos diagrams integrate all mutation information of primary tumors and BMs. Circle 1: Chromosomes; Circle 2: Purple dots represent genome SNP density; Circle 3: Blue dots represent the distribution of genome InDel density; Circle 4: CNV distribution, red lines represent copy number amplification, blue lines represent copy number deletion, green lines represent normal copy number; Circle 5: SV distribution, supporting reads ≥ 10 are displayed. Interchromosomal translation (brown); Intrachromosomal translocation (blue); Insertion (orange); Deletion (crimson); Duplication (light purple) and Inversion (green).

Supplementary Figure 3
**(A)** EGFR and ALK immunohistochemical staining of primary tumor and BMs in Case 3. **(B)** H&E staining, EGFR and ALK immunohistochemical staining of the primary tumor and paired BM in Case 5. **(C, D)** H&E staining of primary lung tumors and BMs. Scale bar = 100μm

Supplementary Figure 4
**(A, B)** The density of CD4+ T cells in primary lung tumors and paired BMs. **(C)** Comparison of the density of CD8+ T cells infiltration in the MUC16 mutant and wild-type subgroup. Abbreviations: LUAD. Lung adenocarcinoma; TCGA. The Cancer Genome Atlas. Scale bar = 50μm

Supplementary Figure 5
**(A, B)** CD34 immunohistochemical staining evaluates the heterogeneity of MVD between primary tumors and BMs in Case 3 and Case 7, respectively. The lower panel is the magnified images of the black frames (the upper panel). **(C)** Comparison of MVD between matched primary lung tumors and BMs.
