# Supplementary figures and images for: Heterogenous profiles between primary lung cancers and paired brain metastases reveal tumor evolution
Source: Front Oncol. 2023 Jun 13;13:1026099. doi: 10.3389/fonc.2023.1026099 (PMC10293929; doi:10.3389/fonc.2023.1026099)

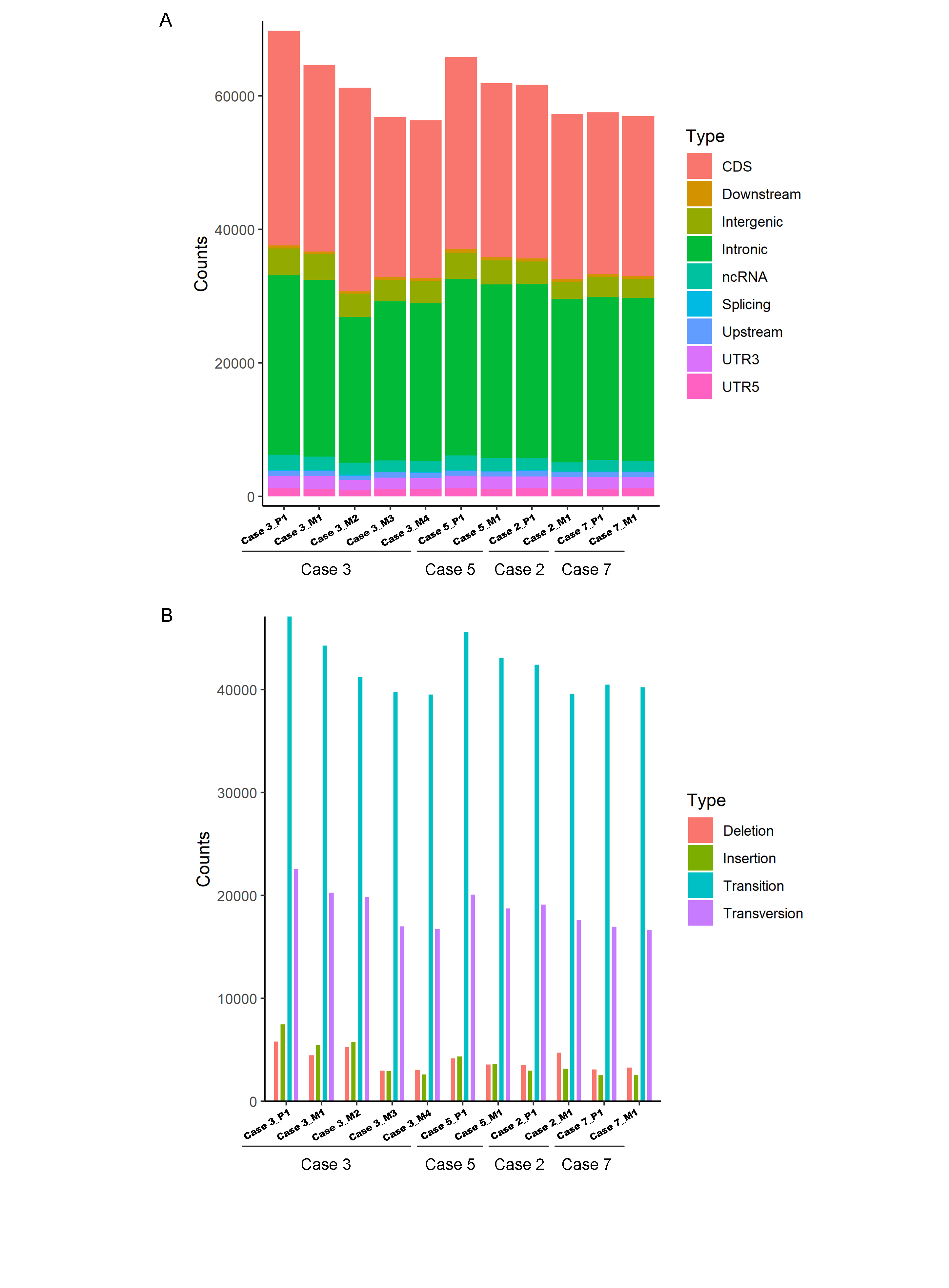

Supplement: Supplementary Figure 1 — (A) The distribution of various sites of SNP&InDel mutations in primary lung cancers and BMs; (B) Overall distribution of mutation types. [file Image_1.jpeg]

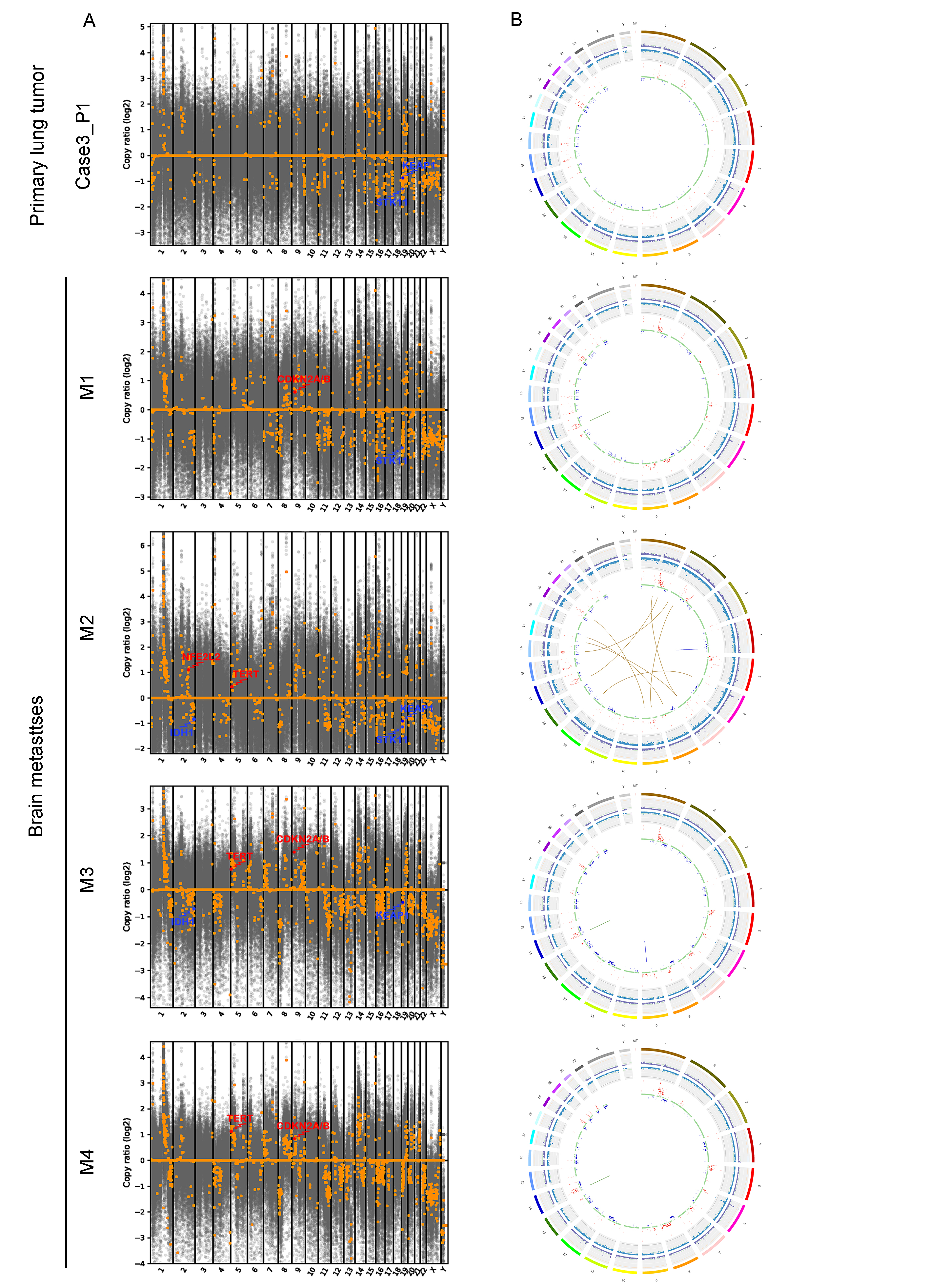

Supplement: Supplementary Figure 2 — (A) Landscapes of copy number variation. (B) Circos diagrams integrate all mutation information of primary tumors and BMs. Circle 1: Chromosomes; Circle 2: Purple dots represent genome SNP density; Circle 3: Blue dots represent the distribution of genome InDel density; Circle 4: CNV distribution, red lines represent copy number amplification, blue lines represent copy number deletion, green lines represent normal copy number; Circle 5: SV distribution, supporting reads ≥ 10 are displayed. Interchromosomal translation (brown); Intrachromosomal translocation (blue); Insertion (orange); Deletion (crimson); Duplication (light purple) and Inversion (green). [file Image_2.jpeg]

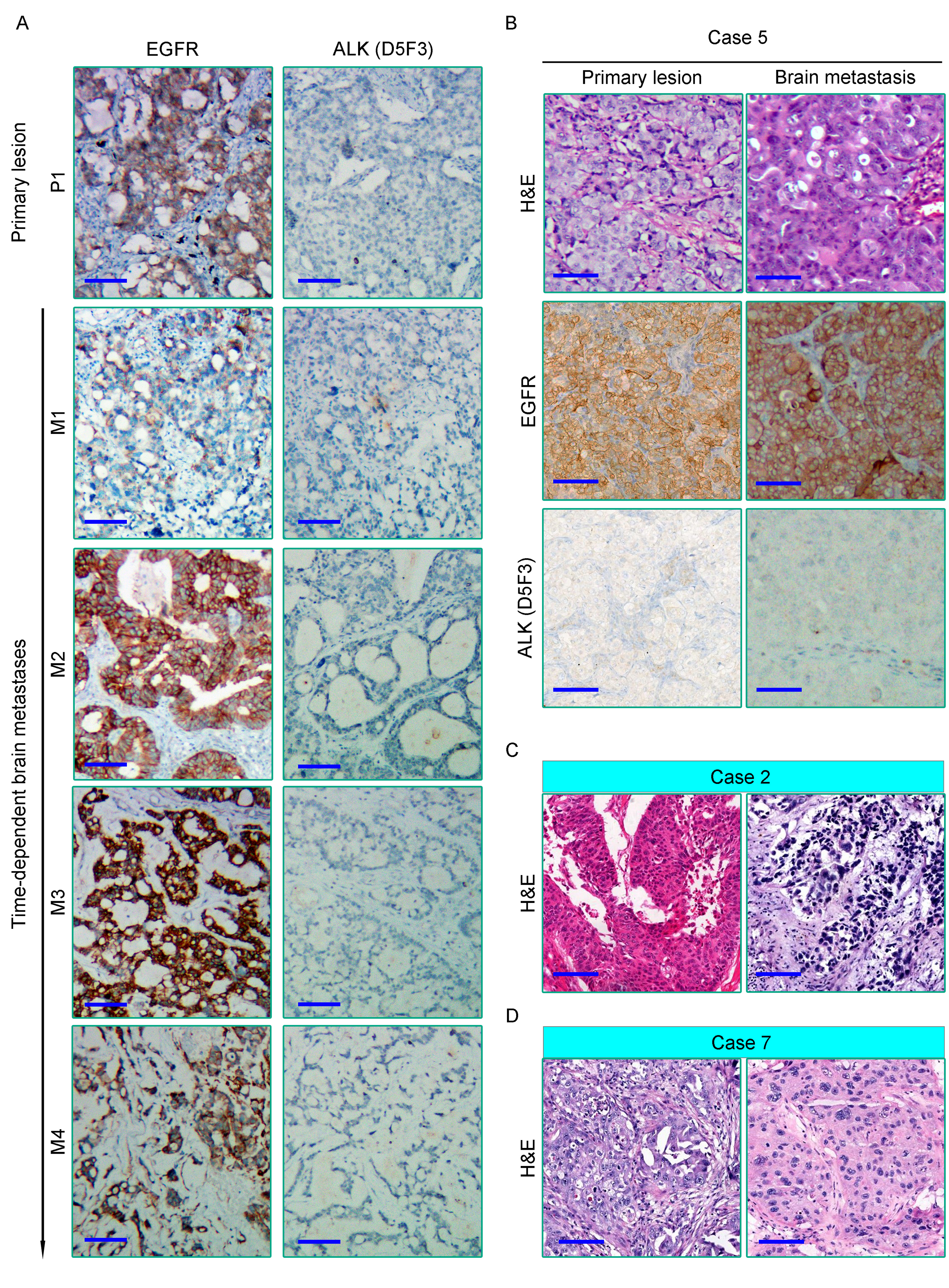

Supplement: Supplementary Figure 3 — (A) EGFR and ALK immunohistochemical staining of primary tumor and BMs in Case 3. (B) H&E staining, EGFR and ALK immunohistochemical staining of the primary tumor and paired BM in Case 5. (C, D) H&E staining of primary lung tumors and BMs. Scale bar = 100μm [file Image_3.jpeg]

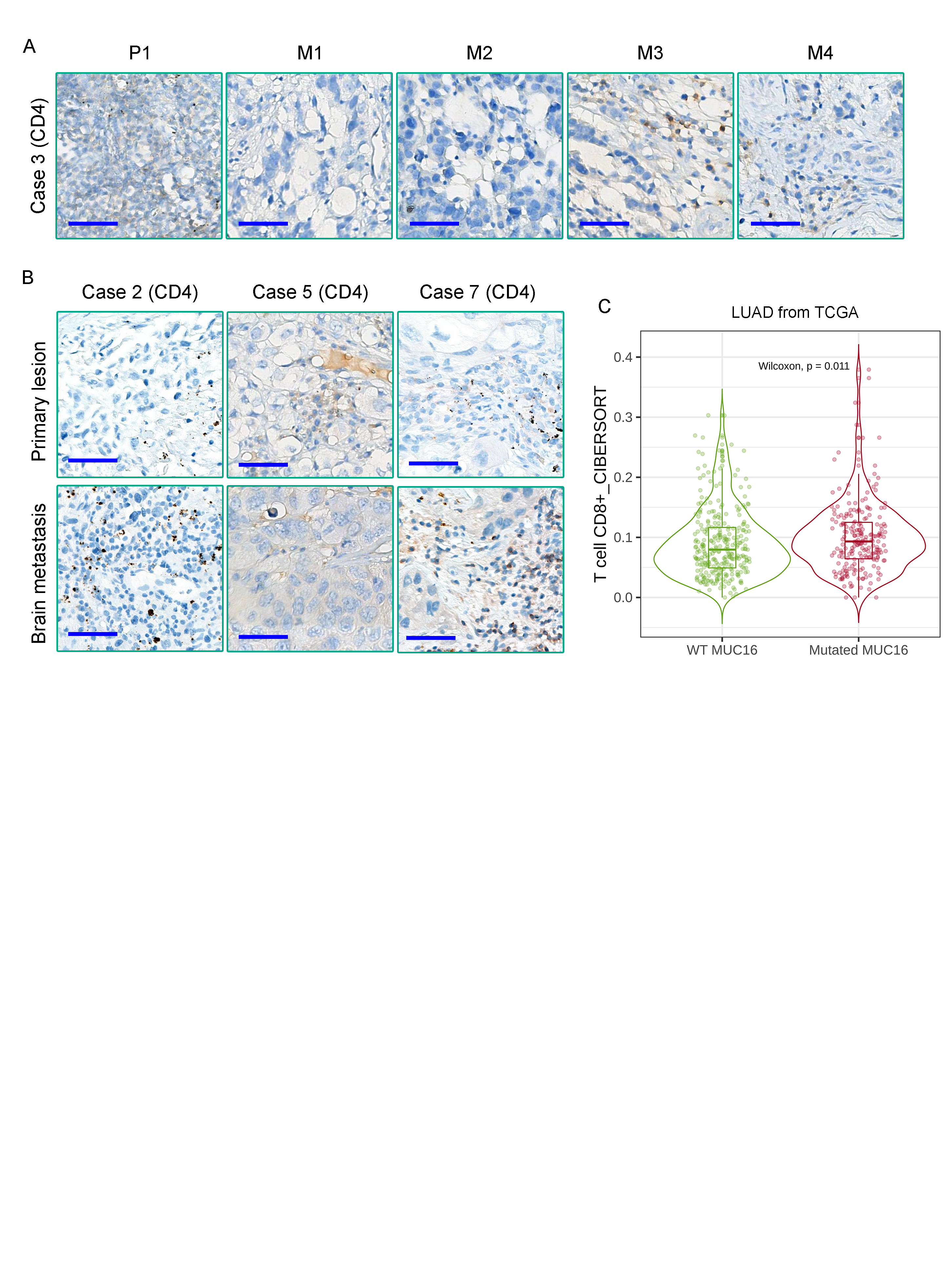

Supplement: Supplementary Figure 4 — (A, B) The density of CD4+ T cells in primary lung tumors and paired BMs. (C) Comparison of the density of CD8+ T cells infiltration in the MUC16 mutant and wild-type subgroup. Abbreviations: LUAD. Lung adenocarcinoma; TCGA. The Cancer Genome Atlas. Scale bar = 50μm [file Image_4.jpeg]

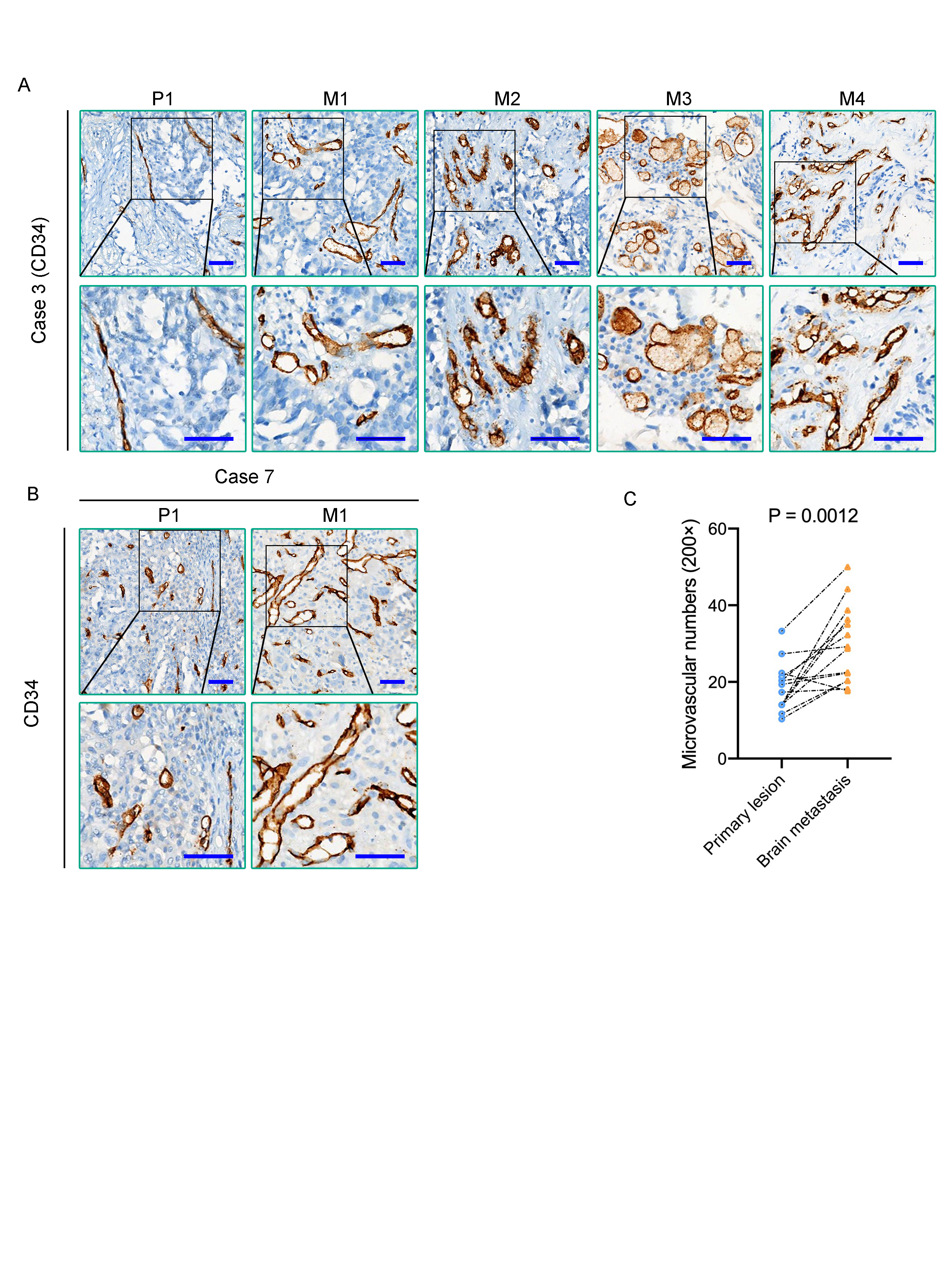

Supplement: Supplementary Figure 5 — (A, B) CD34 immunohistochemical staining evaluates the heterogeneity of MVD between primary tumors and BMs in Case 3 and Case 7, respectively. The lower panel is the magnified images of the black frames (the upper panel). (C) Comparison of MVD between matched primary lung tumors and BMs. [file Image_5.jpeg]
