# Supplementary material for: Heterogenous profiles between primary lung cancers and paired brain metastases reveal tumor evolution
Source: Front Oncol. 2023 Jun 13;13:1026099. doi: 10.3389/fonc.2023.1026099 (PMC10293929; doi:10.3389/fonc.2023.1026099)
Supplement: Supplementary file 6 [file Table_1.docx]

Table S1. Unique genetic signatures identified in BMs.

| **Case No.** | **Genomic mutations** | **Copy number variations** |
| --- | --- | --- |
| Case 3 | | |
| M1 | RB1, SET, TP53, PDGFRA | CDKN2A/B |
| M2 | CTNND2, TP53, ALK, SET, JAK2, PDGFRA | IDH1, TERT, NFE2L2 |
| M3 | RB1, SET, TP53 | IDH1, TERT, CDKN2A/B |
| M4 | JAK2, TP53 | TERT, CDKN2A/B |
| Case 5 | SET, ROS1, AKT3, CTNND2 | MET, KRAS, CIC |
| Case 2 | AKT2, TERT, BRAF, MAP3K1 | KRAS |
| Case 7 | NFKB2 | - |
