# Supplementary material for: Heterogenous profiles between primary lung cancers and paired brain metastases reveal tumor evolution
Source: Front Oncol. 2023 Jun 13;13:1026099. doi: 10.3389/fonc.2023.1026099 (PMC10293929; doi:10.3389/fonc.2023.1026099)
Supplement: Supplementary file 7 [file Table_2.docx]

Table S2. The molecular phenotypic characteristics of primary lesions and paired BMs.

| **Case No.** | **Exp.ID** | **Molecular phenotype** | |
| --- | --- | --- | --- |
|  |  | **Primary lesion** | **Metastatic lesion** |
| 1 | LC_001 | CK5/6+, TTF1-, EGFR+, PD-L1+, CD4+, CD8+, CD34+ | TTF1-, EGFR+, GFAP-, PD-L1+, CD4+, CD8+, CD34+ |
| 2 | LC_002 | EGFR+, PD-L1+, CD4+, CD8+, CD34+ | GFAP-, PD-L1+, CD4+, CD8+, CD34+ |
| 3 | LC_003 | EGFR+, ALK(D5F3)-, PD-L1+, CD4-, CD8+, CD34+ | CK7+, CK20-, GFAP-, Villin-, S100-, TTF1+, PE10+, CDX2-, CD10-, CEA+, ALK(D5F3)-, PD-L1+, CD4-, CD8+, CD34+ |
|  | LC_004 |  | GFAP-, S100-, CK7+, Villin-, TTF1+, ALK(D5F3)-, PD-L1+, CD4-, CD8+, CD34+ |
|  | LC_005 |  | TTF1-, CK7+, CDX2-, Villin-, Ki67+(70%), ALK(D5F3)-, PD-L1+, CD4-, CD8+, CD34+ |
|  | LC_006 |  | CK7+, CK20-, Villin-, TTF1-, EGFR-, P53+, ALK(D5F3)-, PD-L1+, CD4-, CD8+, CD34+ |
| 4 | LC_007 | CK7+, TTF1+, EGFR+, PD-L1+, CD4+, CD8+, CD34+, | CK7+, TTF1+, NapsinA+, P40-, CK5/6-, ER-, PR-, Ki67+, GFAP-, NF-, EMA-, S100-, CK7+, CK20-, Villin-, PD-L1+, CD4+, CD8+, CD34+ |
| 5 | LC_008 | AE1/AE3+, Syn-, CD56-, TTF1+, NapsinA+, EGFR+, ALK(D5F3)-, PDL1+, CD4-, CD8+, CD34+ | AE1/AE3-, TTF1+, NapsinA-, CD56-, Syn-, Ki67+, EGFR+, ALK(D5F3)- , PDL1+, CD4-, CD8+, CD34+ |
| 6 | LC_010 | EGFR+, TTF1+, ALK(D5F3)+, PD-L1+, CD4+, CD8+, CD34+, | CK7+, CK5/6-, NapsinA-, TTF1-, P40-, GFAP+, ALK(D5F3)+, Ki67+, PD-L1+, CD4+, CD8+, CD34+ |
|  | LC_011 |  | CK7+, TTF1-, NapsinA-, AE1/AE3+, GFAP+, ALK(D5F3)+, Ki67+, PD-L1+, CD4+, CD8+, CD34+ |
| 7 | LC_013 | AE1/AE3+, GFAP-, CK7+, TTF1+, EMA+, EGFR+, ALK(D5F3)-, PD-L1+, Ki67+, CD4-, CD8+, CD34+ | CK7+, EGFR+, ALK(D5F3)-, PD-L1+, Ki67+, TTF1+, CD4-, CD8+, CD34+ |
| 8 | LC_014 | TTF1+, NapsinA+, EGFR+, PD-L1+, CD4+, CD8+, CD34+ | CK7+, TTF1-, NapsinA-, CK20-, CK19+, Ki67+(25%), PD-L1+, CD4-, CD8+, CD34+ |
| 9 | LC_015 | TTF1+, CK7+, EGFR+, PDL1+, CD4-, CD8+, CD34+ | GFAP-, S100-, NF-, NCE-, Syn-, CK20-, CK7+, Villin-, TTF1+, CEA+, Ki67+(20%), PD-L1+, CD4+, CD8+, CD34+ |
| 10 | LC_016 | NapsinA+, EGFR+, TTF1+, ALK(D5F3)+, PD-L1+(20%), CD4+, CD8+, CD34+ | CK7+, TTF1+, NapsinA+, CEA+, Vimentin+, PD-L1+(5%), ALK(D5F3)+, Ki67+(10%), CD4-, CD8+, CD34+ |
